# Current State of Modeling Human Psychiatric Disorders Using Zebrafish

**DOI:** 10.3390/ijms24043187

**Published:** 2023-02-06

**Authors:** Fabiano V. Costa, Tatiana O. Kolesnikova, David S. Galstyan, Nikita P. Ilyin, Murilo S. de Abreu, Elena V. Petersen, Konstantin A. Demin, Konstantin B. Yenkoyan, Allan V. Kalueff

**Affiliations:** 1 Institute of Translational Biomedicine, St. Petersburg State University, 199034 St. Petersburg, Russia; 2 Neurobiology Program, Sirius University of Science and Technology, 354340 Sochi, Russia; 3 Almazov National Medical Research Centre, Neuroscience Group, Institute of Experimental Medicine, Ministry of Healthcare of Russian Federation, 197341 St. Petersburg, Russia; 4 Granov Russian Research Center of Radiology and Surgical Technologies, Laboratory of Preclinical Bioscreening, Ministry of Healthcare of Russian Federation, 197758 Pesochny, Russia; 5 Laboratory of Cell and Molecular Biology and Neurobiology, Moscow Institute of Physics and Technology, 141703 Moscow, Russia; 6 Neuroscience Group, Ural Federal University, 620002 Yekaterinburg, Russia; 7 Neuroscience Laboratory, COBRAIN Center, Yerevan State Medical University Named after Mkhitar Heratsi, Yerevan 0025, Armenia; 8 Department of Biochemistry, Yerevan State Medical University Named after Mkhitar Heratsi, Yerevan 0025, Armenia

**Keywords:** *Danio rerio*, animal modelling, translational biopsychiatry, psychiatric disorders

## Abstract

Psychiatric disorders are highly prevalent brain pathologies that represent an urgent, unmet biomedical problem. Since reliable clinical diagnoses are essential for the treatment of psychiatric disorders, their animal models with robust, relevant behavioral and physiological endpoints become necessary. Zebrafish (*Danio rerio*) display well-defined, complex behaviors in major neurobehavioral domains which are evolutionarily conserved and strikingly parallel to those seen in rodents and humans. Although zebrafish are increasingly often used to model psychiatric disorders, there are also multiple challenges with such models as well. The field may therefore benefit from a balanced, disease-oriented discussion that considers the clinical prevalence, the pathological complexity, and societal importance of the disorders in question, and the extent of its detalization in zebrafish central nervous system (CNS) studies. Here, we critically discuss the use of zebrafish for modeling human psychiatric disorders in general, and highlight the topics for further in-depth consideration, in order to foster and (re)focus translational biological neuroscience research utilizing zebrafish. Recent developments in molecular biology research utilizing this model species have also been summarized here, collectively calling for a wider use of zebrafish in translational CNS disease modeling.

## 1. Introduction

Psychiatric disorders are highly prevalent brain illnesses that represent a major urgent, unmet biomedical problem [1,2,3,4,5]. Their prevention and treatment involves three main challenges: to identify a genotype associated with the disorder in question, to characterize molecular pathology underlying each disorder, and to develop novel efficient therapies [6]. Unlike clinically robust neurological disorders, such as Alzheimer’s and Parkinson’s diseases, most psychiatric pathologies do not have detectable pathobiological signs (e.g., neuronal loss or protein aggregation), hence heavily relying on behavioral and cognitive phenotypes for correct diagnostics [1]. Although complex genetic bases of human psychiatric disorders and their clinical heterogeneity make it impossible to fully mimic clinical conditions using laboratory animals [2,7], such experimental models represent an increasingly important tool in translational research of various pathogenic aspects of psychiatric disorders [8,9].

Zebrafish (*Danio rerio*) are small freshwater teleost fish that have recently become a powerful model organism in translational neuroscience research [10]. These fish are currently widely used in major universities and research centers worldwide, bringing to neuroscience research both reliability and high throughput [10]. Multiple advanced genetic tools (e.g., CRISPR-Cas9 or transcription activator-like effector nucleases, TALENS) [11], as well as optogenetics-based [12,13] and neuroimaging methods [14], have also been successfully applied to zebrafish models. Furthermore, zebrafish display robust, well-defined, context-specific and complex behaviors in all major central nervous system (CNS) domains, which are generally evolutionarily conserved and strikingly parallel to those in rodents and humans [15].

Recognizing multiple challenges in translational psychiatric research using zebrafish models, here we critically review recent developments in this field, and highlight key topics for further in-depth consideration, in order to foster and better (re)focus translational neurobiological research utilizing zebrafish. Recent developments in molecular biology research using this model species have also been summarized here, collectively calling for a wider use of zebrafish in CNS disease modeling.

## 2. Current State of Studying Zebrafish Model of Psychiatric Disorders

**Figure 1:**
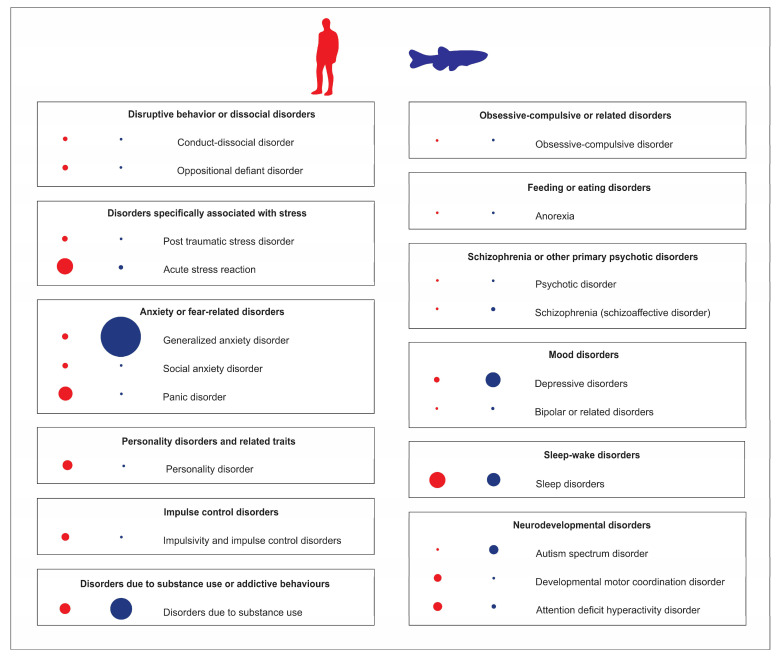
Analyses of Pubmed publications using zebrafish as an animal model of various human psychiatric disorders, compared to their clinical prevalence in adults. Blue dots (on the right) represent the relative number of zebrafish publications on specific psychiatric disorders, red dots (on the left) denote their relative clinical prevalence. The dot size reflects the relative frequency of each parameter.

Modern classification of human psychiatric disorders is typically based on the International Classification of Diseases and Related Health Problems (ICD-11) and the Diagnostic and Statistical Manual of Mental Disorders (DSM-5, Figure 1). Since the global prevalence of major human psychiatric disorders (also see Supplementary Table S1) reflects their relative clinical and societal importance, a major challenge for zebrafish-based CNS disease modeling is to ensure that clinical prevalence/importance of CNS disorders is adequately reflected in current trends of zebrafish research. Addressing this question, our analyses of current trends in zebrafish literature in PubMed database for specific CNS disorders (Figure 1) resulted in several considerations. Notably, drug-induced brain disorders are highly prevalent, societally and clinically important illnesses, whose occurrence rose by 45% in the last decade, making them a major global health problem [16]. Although cannabis remains by far the most commonly used/abused drug, opioids present the greatest harm to the health of users [16]. Importantly, zebrafish possess all opioid [17,18,19], cannabinoid [20], and monoaminergic systems [21,22,23] that play a key role in drug-induced psychiatric disorders.

However, as shown in Figure 1, the most studied psychiatric disorder in zebrafish models is generalized anxiety disorder, which is likely heavily overrepresented in the zebrafish literature (44%) compared to its estimated 7% global clinical prevalence. On the one hand, zebrafish are indeed a sensitive and efficient model system for studying anxiety disorders, with a set of well-described anxiety-like behaviors and easily applicable experimental protocols and assays (e.g., novel tank test; NTT, light dark box test; LDT, open field test; OFT, predator exposure test) that, like their well-established rodent counterparts, typically employ novelty-based or fear-based paradigms (see [24,25,26,27] for a comprehensive review). Paralleling behavioral endpoints, neurochemical and endocrine (e.g., cortisol) biomarkers of zebrafish anxiety are also widely used in modeling affective pathogenesis in fish [24,25,26,27]. Multiple clinically active anxiolytic and anxiogenic drugs also potently modulate anxiety-like behaviors in zebrafish, and can be reliably assessed in fish behavioral assays mentioned above [28]. Additionally, most zebrafish brain regions are well described, having major important neuroanatomical homologues for key mammalian brain regions that control behavior [29]. For example, zebrafish possess medial pallium and habenula [30,31,32], homologous to several brain structures responsible for anxiety-like behaviors in humans.

The activation of zebrafish neuroendocrine hypothalamic-pituitary-interrenal (HPI) axis, physiologically homologous to human hypothalamic-pituitary-adrenal (HPA) axis, triggers the release of cortisol [33], further strongly supporting the use of these fish for studying anxiety spectrum disorders and their pathophysiology [34].

However, there are also clear limitations in zebrafish use to study stress pathobiology. For example, since it is impossible to obtain a sufficient amount of blood without euthanizing the animal (due its small size), the long-term monitoring of stress responses from blood samples is problematic [35]. Moreover, fish live in an aquatic environment where they constantly release hormones and metabolites related to stress responses [36]. Thus, unlike terrestrial vertebrates and humans, zebrafish continuously absorb these substances, which in turn may also play a role in modulating their stress responsivity. Nevertheless, although this factor may contribute to some discrepancy in physiological and behavioral responses to stress in fish vs. humans, there are also multiple well-described and simple experimental protocols to access acute stress responses in zebrafish [34,37]. Thus, despite these environmental differences, the overall neuroendocrine similarity between zebrafish and humans, together with well-described behavioral stress protocols, collectively make zebrafish a reliable model to study stress-related brain disorders.

On the other hand, acute stress studies in zebrafish also present some discrepancies in the existing literature. For example, an analysis of acute stress reaction is currently underrepresented in zebrafish studies (3%), compared to their clinical prevalence of 15% (Figure 1). Described by ICD-11 as “development of transient emotional, somatic, cognitive, or behavioural symptoms as a result of exposure to an event or situation of an extremely threatening or horrific nature”, acute stress reaction differs from post-traumatic stress disorder (PTSD), as the former usually subsides within days after stress, whereas the latter persists for several weeks [38].

Such phenotypic variance highlights several important factors for CNS disease modelling using zebrafish. Consider, for example, a marked difference in the numbers of clinical cases of acute vs. delayed acute severe stress reactions that may correspond to underlying individual differences in stress responsivity between patients, with some subjects being more susceptible to a stress exposure (and developing longer-lasting CNS disturbances) than the others. This aspect is critical for valid CNS disease modelling, since some animals as well may not develop long-lasting deficits without genetic or environmental triggers. Furthermore, the existence of CNS pathologies that are induced by the same factor(s), but occur at different time frames, necessitates detailed phenotyping of the models at different time points. For this, zebrafish may represent a valuable model for time-dependent phenotyping by having a relatively long lifespan (~4 years) with a prolonged duration of the adult state. Such approach has already been implemented in stress studies assessing complex dynamics of behavioral and neurochemical phenotypes in zebrafish affective disorders [39].

Although sleep disorders, especially insomnia, are among the most common human psychiatric disorders (Figure 1), with global prevalence between 10 and 60%, this group is remarkably underrepresented in current zebrafish research, with only 1% of studies exploring insomnia-related behavior. Note, however, that circadian rhythm disorders are rather overrepresented in the zebrafish literature, with 10% of zebrafish studies (vs. 3% of the former global prevalence in humans) [40]. Importantly, zebrafish possess a well-described behavioral sleep state (e.g., circadian-regulated periods of reversible immobility associated with an increased arousal threshold [41,42,43] and sleep rebound in response to sleep deprivation [41,42,44]), as well as neuronal signatures of sleep [45]. Additionally, major neurocircuits responsible for the regulation of sleep–wake cycle are subcortical and evolutionarily conserved across vertebrate species, including zebrafish [41,44,46]. Thus, while some sleep disorders may be difficult or even impossible to recapitulate in zebrafish (e.g., apnea), zebrafish emerge as an important tool to investigate sleep disorders (and related psychiatric disorders), especially insomnia.

Furthermore, because many psychiatric disorders have strong genetic bases [47,48,49,50], it is logical to apply genetic modelling to recapitulate disorder-specific symptoms, and to utilize various omics-based tools to study complex molecular cascades associated with neuropsychiatric disorders. However, while many neuropsychiatric disorders are polygenic in nature [51,52], genome-wide associations studies (GWAS) often report multiple polymorphisms even within a single gene that contribute to the observed clinical phenotypes [53,54,55], further complicating genetic modelling of such conditions. Similarly, multiple transcriptomic studies show altered expression of various brain CNS genes in neuropsychiatric disorders [56,57,58,59,60,61]. Thus, it is logical to consider combining several genetic mutations to properly model specific CNS disorders of interest.

One such genetic animal model targets Alzheimer’s disease to induce a more severe experimental pathogenesis in mice that closely mimics human conditions [62]. For example, 5xFAD mice overexpress two transgenes combining five mutations—Swedish K670N/M671L, London V717I, and Florida I716V *hAPP* mutations with M146L and L286V *hPSEN1* mutations [63], whereas 3xTg mice harbor Swedish K670N/M671L, M146L *hPSEN1,* and P301L h*MAPT* mutations [64]. However, to the best of our knowledge, there is a current lack of zebrafish studies with polygenic genetic modelling of psychiatric conditions. For example, one may consider to knockout one of the glutamate receptor (*gr*) copies, *slc6a4a* (one of serotonin transporter copies), and a key interleukin (IL), *il10* gene, hence breaking proper HPI/HPA axis signaling, inducing monoamines disbalance, and increasing inflammatory response at the same time. Likewise, combining *disc1* (disrupted in schizophrenia-1), *nrg1* (neuregulin-1), *akt1* (AKT serine/threonine kinase 1), and/or *dtnbp1a/b* (dysbindin-1 homologues) mutations may eventually lead to interesting models of schizophrenia-like conditions in fish. Clearly, albeit rather underdeveloped in fish, such polygenetic models are critically important and translationally relevant, as they may better reflect “true” CNS pathogenesis occurring in human psychiatric disorders.

Moreover, some translational studies may examine the molecular alterations in other (e.g., behavioral and pharmacological) animal models using omics-related tools (e.g., RNA-seq) to find evolutionally conserved biomarkers of CNS disorders that may be crucial for neuropathogenesis in both humans and zebrafish. For example, a widely used model of affective pathology in rodents and zebrafish, the chronic unpredictable stress (see further), reveals multiple transcriptomic changes in the brain that parallel deficits seen in human CNS diseases [39,65]. Specifically, chronic unpredictable stress in zebrafish induces differential expression of genes involved in the inflammation/cytokine-related signaling pathways, mitogen-activated protein kinase (MAPK) signaling, and receptor tyrosine kinases, including signal transducer and activator of transcription (*stat*) *1b* and *4*, interleukin 21 receptor (*il21r*), janus kinase 3 (*jak3*), and suppressor of cytokine signaling (*socs*) *1a,* all long associated with clinical affective pathology and inflammation [39,65].

Furthermore, such chronic stress alters the expression of multiple endocrine and signaling receptor-related genes, further paralleling human pathology [39]. Interestingly, *serpini1*^-/-^ knockout zebrafish display anxiety-like behavior, with the expression of closely related genes (e.g., *socs1a* and *sagb*) altered based on RNA-seq analysis, supporting their involvement in affective pathology [66]. At the same time, very few such molecular studies have been conducted on other psychiatric disorders (beyond anxiety spectrum) in zebrafish models, clearly necessitating further analyses.

Combining genetic, epigenetic, environmental, behavioral or drug-based experimental models to better recapitulate disorders pathogenesis, also seems timely. For instance, as already noted, only few subjects develop PTSD following a severe acute stress exposure, due to specific molecular or environmental risk factors. The gene-environment interactions (GxE) and sex-environment interactions (SxE) have recently gained an increased recognition in psychiatric disorder modeling [67,68]. GxE and other similar interactions reflect how individual genotypes influences the sensitivity to environmental stimuli that trigger CNS pathogenesis, and their use is highly beneficial for successful experimental modelling of brain disorders [69,70,71,72,73]. For example, using serotonin transporter knockout (5*htt a* or *b*) in combination with severe stress exposure may help recapitulate clinical data linking human serotonin transporter *5HTT* genetic polymorphisms to affective disorders [74,75]. Likewise, combining schizophrenia-related models (e.g., *disc1* knockout) with prenatal inflammatory exposure (e.g., Poly I:C or LPS) and early life stress, may also be relevant to modeling schizophrenia pathogenesis [76].

Another important factor to consider is that aberrant phenotype itself may affect the environment to which an individual is exposed, without direct effects on disorder pathogenesis *per se* [68]. For example, children affected by a neuropsychiatric disorder (e.g., autism or depression) may be socially isolated by their peers, further impairing their development and behaviors [77]. Taking together, such complex interplay between multiple genetic and environmental factors necessitates novel conceptual and methodological approaches that will target multiple pathogenetic factors in order to create more valid and efficient models of human psychiatric disorders. In general, current zebrafish models usually lack such integrative approaches, clearly calling for further studies in this direction.

## 3. Case in Point: Molecular Approaches to Modeling CNS Disorders in Zebrafish—Insights from Chronic Unpredictable Stress

Depression is the leading cause of human disability worldwide [78,79], representing a severely debilitating neuropsychiatric disorder that is highly heterogeneous in pathogenesis, clinical signs, and comorbidities [80,81]. Depression treatment is often complicated due to its treatment-resistance nature, frequent recurrence, and common comorbidity with other clinical disorders, both neural and non-neural [82,83,84]. Animal models are widely used as an indispensable tool to probe depression pathogenesis, including using highly invasive experimental manipulations [81]. For obvious ethical and practical reasons, most studies aiming to recapitulate depression pathogenesis utilize rodent models [85,86,87]. However, zebrafish have also emerged recently as an important complementary tool to model a wide range of affective pathologies, including depression [88,89]. Zebrafish attract the growing attention in the field, given their multiple advantages, including easy genetic manipulations, simple behavioral phenotypes, conserved CNS morphology as well as high-throughput capabilities, allowing for fast antidepressant drugs screening [90,91,92]. There is also an expectation that the use of zebrafish may enable targeting “core”, evolutionarily conserved pathological cascades underlying affective pathogenesis in depression [93,94].

Various experimental depression models have been proposed in animals, and some of them are already available in zebrafish [93]. Such models can be generally divided into three main categories, involving physical (e.g., behavioral and environmental), pharmacological (e.g., small molecules and inflammatory agents), or genetic (e.g., gene knockout or silencing) manipulations. Given their relative simplicity, and because stress is one of the key factors in depression pathogenesis, chronic unpredictable stress paradigms have become widely popular in rodent and zebrafish studies [95,96]. Such models typically involve daily exposure of animals to varying (hence unpredictable) stressors for weeks (e.g., ranging from one [97] to as long as 12 weeks [98] in zebrafish) to induce depression- and anxiety-like state. 

On the one hand, chronic unpredictable stress is one of most widely used stress models in both rodents and zebrafish [98]. On the other hand, vague description of the models’ battery of stressors often results in high variability of data, further complicated by the fact that different groups often use different stressors, age groups, sexes, and longevity aspects, thus collectively impeding reproducibility of such analyses. The latter, however, is particularly important in terms of their relevance to clinical data, where chronic stress typically lasts much longer than few weeks, making many animal-based chronic stress models less relevant translationally. As a result, while being studied for a relatively long period of time, molecular findings from these chronic stress models remain rather limited in both taxa, especially in terms of their translatability to clinical data.

In rodents, chronic unpredictable stress markedly affects the expression of various brain genes. For example, 7-week stress lowers the expression of several glutamate receptor subunits (*Grin2a*, *Grin2b*, *Grin2c*, *Grin3a*, *Gria4*, and *Grm3-8*) in rodent cerebral cortex and amygdala [99], whereas a 5-week stress upregulates hippocampal expression of glutamate transporter genes (*Vglut1* and *Vglut2*) in mice [100]. Such stress can also alter the expression of glutamate transporter genes (*Slc17a6* and *Slc17a8*) in young mice, one increasing, and the other decreasing, CNS expression, respectively [101]. Chronic unpredictable stress can also influence the expression of brain genes related to growth factors and neurogenesis (such as *Bdnf* [102] and *Igf-2* [103]), as well as molecules involved in neuronal signaling (*Ffg*, *Ngf*, *Vegf*, *Egf,* and *Igf-1*) in rodent amygdala [99,104]. However, most transcriptomic studies in rodents do not correlate with monoamine neurotransmitter activity, thus complicating understanding of the effects of serotonergic antidepressants in animal models (and clinically). Furthermore, rodent molecular studies often remain poorly reproducible and do not find confirmation in clinical settings [105,106,107,108,109,110].

Several studies have attempted to assess transcriptomic and other molecular changes in zebrafish brain following chronic unpredictable stress. For instance, 2-week chronic unpredictable stress alters the expression of various genes in the telencephalon, a critical CNS area associated with cognitive and affective functions [65]. Orthologues of two altered zebrafish CNS genes, cyclin-dependent kinase 5 (*cdk5*) and cholinergic receptor nicotinic alpha 7 subunit (*chrna7*), are also involved in learning and memory in mammals, while *draxin* (encoding dorsal inhibitory axon guidance protein) regulates their hippocampal organization and neurogenesis [65]. The Gene Ontology Biological Process “structural molecule activity” is also downregulated for these genes [65]. In contrast, the upregulation of Orange domain-related genes (*her4.2*, *her6*, *her8.2*, *hey1*) and genes of interferon alpha-inducible protein IFI6/IFI27-like proteins (IFI6/IFI27-like; *si:dkey-188i13.7*, *zgc:152791*, *zgc:123068*), occurs in the telencephalon of stressed zebrafish [65]. While the orange domain is a motif present in transcription repressors, and is involved in neurogenesis, the IFI6/IFI27-like protein domain is poorly characterized and has not yet been linked to any biological processes or molecular functions [65]. Clearly meriting further scrutiny, based on zebrafish findings, the latter gene set consists of interferon inducible-like proteins, suggesting some potential neuroinflammation that may be involved in stress CNS effects.

Overall, these studies support a significant role of inflammation in the development of affective disorders across taxa in vertebrates. As already noted, chronic unpredictable stress in zebrafish alters CNS cytokine networks, which can be corrected by fluoxetine treatment [94]. Additionally, recent neurogenomic analyses in zebrafish support the putative link between affective pathogenesis and adhesion G protein-coupled receptors (GPCRs), as well as arrestins, which serve as adaptor proteins to regulate GPCR signaling [94]. Furthermore, genes controlling ubiquitination and deubiquitination may also play a role in the activity of arrestins and in antidepressant treatment, as they have also been altered in the brain of chronically stressed zebrafish [94]. Collectively, these finding parallel clinical and rodent evidence, and suggest that zebrafish affective pathogenesis is associated with both neuroinflammation and neurotransmitter deficits.

Interestingly, genes from IFI6/IFI27-like protein domain (including *zgc:152791)* reduce their CNS expression in zebrafish exposed to 5-week chronic unpredictable stress, and some of them remain reduced even after 1-week antidepressant (fluoxetine) treatment [39]. As already noted, chronic unpredictable stress alters CNS expression of genes related to inflammation, MAPK signaling, and receptor tyrosine kinases (e.g., *stat1b*, *stat4*, *il21r*, *rsad2*, *jak3*, *zap70*, *socs1a*, *ror1,* and *themis*) [39]. Furthermore, chronic unpredictable stress also affects the expression of genes related to cytoskeleton and cell motility (e.g., *myl1*, *myh1.1*, *my6, tnnt2a, tnnt2d,* and *tnnt2a.1*), as well as ubiquitin-related genes [39], which are linked to interferon-associated genes (e.g., *isg15*) that may serve as a molecular hub linking neuroinflammation and cytokine activity to chronic unpredictable stress.

Stress also disrupts CNS expression of genes related to phototransduction (assessed as under-enrichment of the Kyoto Encyclopedia of Genes and Genomes/KEGG *dre04744* pathway), endocrine function (*vtg1*, *vtg2*, *vtg5*), and RNA processing (assessed as enrichment in Gene Ontology database *GO:0006397 mRNA processing*, *GO:0003735 structural constituent of ribosome* and KEGG *dre03010 ribosome* pathways). Notably, treating stressed fish with a conventional, clinically active antidepressant drug fluoxetine normalizes the expression of many of CNS genes affected by chronic unpredictable stress, particularly those related to cytokine activity. Overall, these findings suggest that chronic unpredictable stress and fluoxetine treatment exert complex effects on CNS gene expression in zebrafish models. However, as already noted, various studies can rather vaguely correspond to each other, and also between model species [111], hence necessitating novel approaches to better translate zebrafish and rodent molecular data into clinical setting.

In general, depression remains relatively understudied in zebrafish (also see further), calling for further development of CNS disease models that specifically target core molecular cascades, thereby enabling mimicking disorder pathogenesis per se, unlike much less specific, traditional behavioral models. While genetic vulnerability has a pronounced effect on depression pathogenesis [112], genetic analyses in human often fail to find reproducible and reliable genetic loci associated with depression [113]. Indeed, only one zebrafish genetic model of depression-like state is currently available, developed a decade ago, the *gr^s357^* zebrafish line with non-functional glucocorticoid receptor (GR) [114,115]. Adult *gr^s357^* zebrafish display pronounced anxiety-like behavior, whereas fluoxetine exposure efficiently rescues it, without affecting the levels of corticotropin-releasing hormone, *serta* and *gr* CNS expression [115].

Another potentially useful strategy in zebrafish CNS disease modeling can involve the inhibition of RNA translation using small interference RNA (siRNAs). While non-specific inhibition of the microRNA pathway may disrupt normal mRNA processing during early zebrafish development [116,117], recent evidence suggests using siRNAs to inhibit target RNA translation in adult zebrafish [118,119]. However, there are yet no studies that would model affective and other CNS pathogenesis in zebrafish using siRNAs.

**Figure 2:**
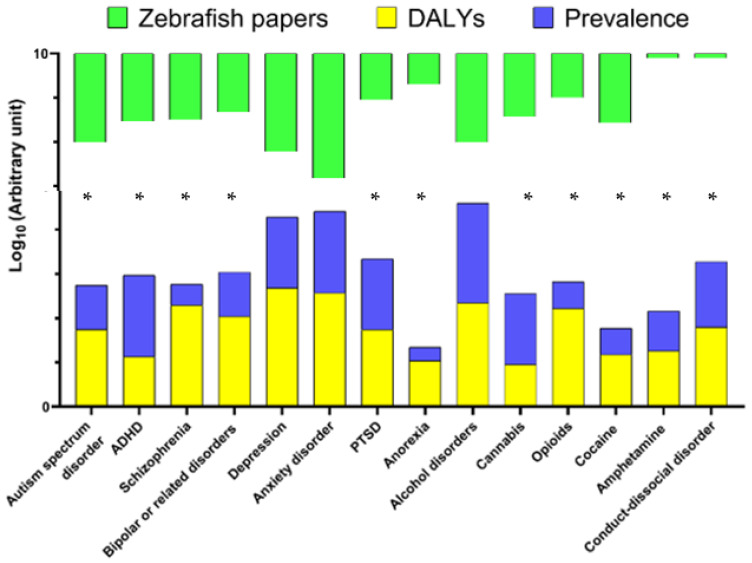
Graphical representation of the number of publications using zebrafish as animal model for various CNS disorders relative to their clinical prevalence in adults, and global burden (DALYs). Areas (denoted by asterisks) with bigger gaps between the bars indicate CNS disorders for which the amount of current clinical and zebrafish evidence does not match, hence meriting further translational modeling efforts.

Finally, while chronic unpredictable stress has a generally good face, construct, and predictive validity as a model of major depression, another affective disorder type related to depression—bipolar disorder—remains remarkably under-represented in zebrafish research (Figure 1 and Figure 2), likely due to its poorly understood pathogenetic mechanisms, multifaceted pathology, and complex clinical phenotype that is difficult to translate into zebrafish behaviors.

## 4. Discussion: Where Next?

Mounting evidence, only briefly discussed here, highlights potential strategic directions for zebrafish-based translational psychiatric research. Firstly, understanding which areas are over- or under-studied, is critical for focusing and refocusing the ongoing zebrafish CNS research. Secondly, some rather fixable factors can further contribute to such imbalances. For example, anxiety, currently grossly over-represented in zebrafish literature (Figure 1), is a frequent psychiatric comorbid condition that shares similar neurological pathways and overlaps with many other psychiatric disorders (e.g., fear, panic, PTSD, and depression) [120,121,122]. Accordingly, attempts to mimic and/or dissect (target separately) such overlapping states in zebrafish behavior may be challenging both practically and conceptually. Moreover, the availability of easy-to-assess anxiety-like behaviors, as well as a wide range of well-established, validated, and reliable experimental protocols, may itself increase the appeal of anxiety research in zebrafish, hence further rising the prevalence of such studies in the literature. The latter, in turn, may encourage new zebrafish laboratories to focus their attention on studying “safe” anxiety phenotypes (rather than probe other, less explored CNS domains) as well, hence further skewing the field.

One problem here is that this may drive the momentum and resources away from studying several other critical affective aspects, such as modeling PTSD- or depression-like states, in zebrafish. Another problem is that simplifying methodological toolbox (e.g., considering the availability of relatively easy and reproducible “popular” anxiety tests in zebrafish) may also promote oversimplification of our interpretation of neurobiological phenomena targeted by various models. For example, while anxiety-like behavior is commonly seen following chronic stress paradigms in zebrafish, it remains unclear whether this affective phenotype represents purely anxiety-like state in fish, a comorbid anxiety/depression condition or, alternatively, mixed “anxiety+depression-like” affective condition triggered by chronic stressors.

In a similar vein, interpreting fish behavior as merely “anxiety” (if affected in standard, well-established anxiety tests) can be misleading, as more thorough analyses may be needed in order to dissect between other related (but not identical) potential fish responses, such as fear-, panic-, aversive avoidance and anhedonia that, depending on the test, may all present as “anxiety”. Likewise, although zebrafish display well-conserved behavior, the complexity of human vs. zebrafish brain makes it difficult to recapitulate the core symptoms of human psychiatric disorders. Thus, this challenge may also explain why some other psychiatric disorders (e.g., insomnia, obstructive sleep apnea, psychotic disorder) are not at all well represented in zebrafish CNS disease modeling field.

Another widespread human brain disorder, relatively well modelled in zebrafish, is autism spectrum disorder (ASD) [123], a complex neurodevelopmental syndrome that manifests as specific deficits in social interactions and as aberrant repetitive behaviors (behavioral perseverations) [80]. Accordingly, most valid zebrafish models of ASD aim to recapitulate social behavior deficits and/or repetitive behavior. In contrast to depression and anxiety models, multiple genetic models that disrupts both behavioral axes are available for ASD modelling in zebrafish [123,124]. For example, missense variations of centrosomal protein 41 gene (*cep41*) that is associated with ASD clinically, disrupt social behavior in zebrafish larvae, also affecting neurodevelopment, axonal growth as well as cranial neural crest cells migration [125]. Similarly, malfunctions of multiple other genes induce social deficits in zebrafish (e.g., in *dyrk1a*, *nr3c2*, and *reln* mutants), sometimes also accompanied by neurological deficits [126,127,128]. Interestingly, *shank3b-/-* zebrafish mutants exhibit both excessive repetitive behaviors, as well as reduced social interaction with developmental deficits, making it one of the most “all-in-one” ASD target in terms of supported clinical endophenotypes in zebrafish [129]. Likewise, genetic knockdown of *syngap1b* or *shank3a* results in common neurodevelopmental phenotype associated with delayed CNS development and motor disruptions [130], and may be relevant to modeling ASD in zebrafish.

A common neurodevelopmental disorder that often overlaps with ASD in terms of symptoms and inherent genetics, attention-deficit/hyperactivity disorder (ADHD), unlike autism, remains poorly studied in zebrafish models [123]. Clinically, ADHD involves difficulty with paying attention and concentrating, that may also accompany ASD [123]. Several genes are known to affect zebrafish hyperactivity [131]. For example, *micall2b* knockdown using morpholino oligonucleotides (MO) leads to hyperactive-impulsive-like behavior in zebrafish that is reversed by a common, clinically used anti-ADHD drug, atomoxetine [132]. Similarly, MO knockdown of *lphn3.1* [133] and mutations in *cntnap2* [134] produce hyperactivity phenotype in zebrafish that may be relevant to ADHD. However, in contrast to affected locomotion, inattention related to ADHD remain understudied in zebrafish, with only one gene (*per1b,* encoding period circadian clock 1b) studied in this regard, whose genetic ablation induces both attention deficits and overt locomotion, hence further corroborating mouse data that link *per1b* to ADHD-like conditions [135].

An important, clinically relevant aspect meriting further consideration is the fact that disease prevalence alone does not represent its overall burden, typically measured as Disability Adjusted Life Years in 100,000 population (DALYs). For example, while schizophrenia has a generally low global prevalence compared to other CNS disorders (Supplementary Table S1), it presents a high DALY value (Table 1, Figure 2), calling for further studies involving animal (e.g., zebrafish) models. Although DALYs may provide a clearer picture here, data on some severe psychiatric disorders, such as developmental motor coordination disorder, are insufficient or presently do not exist in zebrafish. Again, because human psychiatric disorders are highly comorbid, the latter needs better attention in zebrafish studies. Indeed, zebrafish CNS models currently continue to be specific, single disorder-oriented, thus lacking a clinically relevant focus on targeting psychiatric comorbidities.

Thus, we call for making zebrafish models more balanced and consistent with current global trends of clinical prevalence of major psychiatric disorders, in order to make such translational research more biomedically and societally meaningful. This will not only foster further innovative studies of brain pathogenesis, but may also enable the development of novel CNS drugs that the mankind needs. Furthermore, current landscape of human psychiatric disorders rapidly changes, and some disorders (e.g., drug- or stress-related) proliferate more than the others, thereby likely to affect human society more strongly presently than in the past. It is therefore critical that using zebrafish in modeling psychiatric disorders remains focused and up-to-date, following these rapid changes as well, for instance, by paying more attention to the emerging mental health problems (e.g., drug abuse epidemic, Figure 2). Sooner inclusion of zebrafish bioscreens into national and international standards approved and accepted for preclinical drug screening, especially in regard to CNS drugs, may also be warranted.

**Figure 3:**
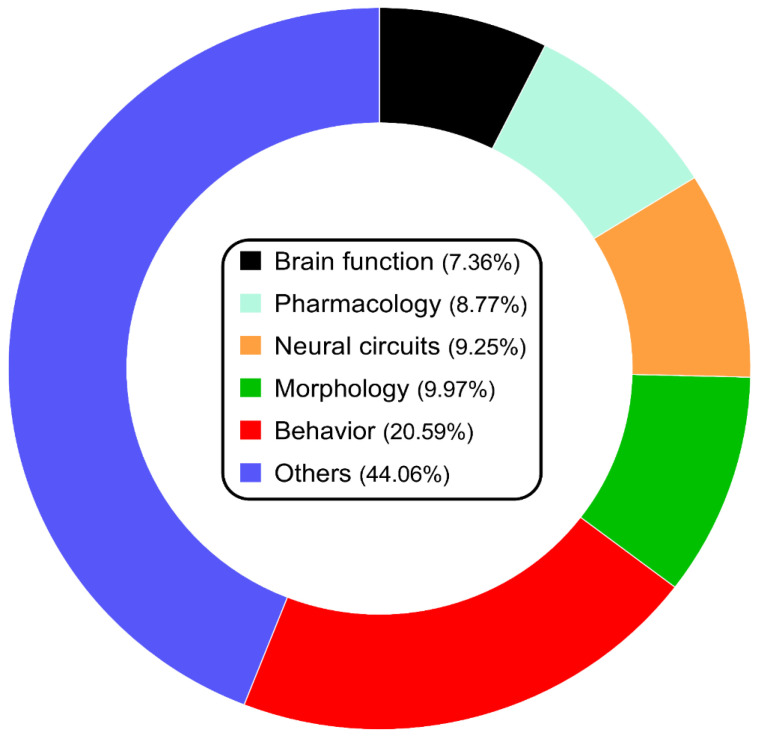
Graphical representation of publications using zebrafish as the animal model in neuroscience (of total taken as 100%) divided into several different major topics.

While proper and reliable clinical diagnosis in psychiatric disorders is essential for their treatment, behavioral studies remain the main component of zebrafish CNS research (Figure 3), helping to develop new techniques to mimic behavioral deficits in fish. As such, future advances in technology will foster the refinement of zebrafish behavioral techniques, for example, generating behavioral fingerprints and sophisticated tools for automated video-based animal tracking [136,137,138], including those based on artificial intelligence (AI) [139], that can bring new insight for a rigorous and thorough animal modeling.

Likewise, albeit not discussed in detail here, not only chronic stress, but also *early-life* (developmental) stress in zebrafish can have long-lasting effects on their behavior and physiology [140]. In line with this, exposure to different types of stressors, including natural psychological and chemical insults, during early development can alter stress responses, and trigger anxiety/depression-like behavior in adulthood, similarly to mammals [141]. Overall, currently even more underrepresented in research trends, studies on early life stress in zebrafish models may provide important insights into how early experiences can shape the development of brain and behavior—a problem that is crucial and key to clinical setting.

Finally, the use of zebrafish to develop novel therapies for human brain disorders can also markedly benefit from conceptual rethinking and synchronizing the very goals of clinical and preclinical studies. Indeed, while the goal of clinical research is to develop safe efficient medications, pre-clinical screening aims to identify the most efficient (but not necessarily the safest) drug that curbs specific disordered phenotypes. From this standpoint, utilizing zebrafish screens may help reconcile these two goals by “clinicizing” outcomes of animal models (i.e., picking zebrafish symptoms more relevant to human disordered phenotypes, or developing models that more closely recapitulate them clinically), yet at the same time paying more attention (than it is done typically) to the safety aspects of drugs tested, in order to be more consistent with the focus of clinical trials. If successful, this can collectively make zebrafish the new gold standard in modeling human psychiatric disorders and their molecular causes, as well as in innovative CNS drug discovery.

## Figures and Tables

**Table 1:** Global burden of psychiatric disorders represented as disability-adjusted life year (DALYs), and current state of zebrafish modeling of these disorders (based on Pubmed papers, as assessed in December 2022).

Human Psychiatric Disorders	DALYs (100.000 Individuals)	Global Prevalence, %	Zebrafish Papers in Pubmed (n)
Autism spectrum disorder	55.66	1	103
ADHD	13.32	7	34
Schizophrenia	195.27	0.3	31
Bipolar or related disorders	109.89	1	21
Depression	480.81	4	167
Anxiety disorder *	370.61	7	665
PTSD	55	4	11
Anorexia	10.96	0.2	5
Alcohol	219.96	18	103
Cannabis	8.92	4	27
Opioids	167.07	0.4	10
Cocaine	14.9	0.4	37
Amphetamine	18.08	0.8	1
Conduct-dissocial disorder	62.97	3	1

* Represents all disorders related to anxiety, such as panic and social disorders. Data from the National Research Council and Institute of Medicine (US).

## Data Availability

Not applicable.

## Supplementary Materials

The supporting information can be downloaded at: https://www.mdpi.com/article/10.3390/ijms24043187/s1.
