# Supplementary material for: Current State of Modeling Human Psychiatric Disorders Using Zebrafish
Source: Int J Mol Sci. 2023 Feb 6;24(4):3187. doi: 10.3390/ijms24043187 (PMC9959486; doi:10.3390/ijms24043187)
Supplement: Supplementary file 1 [file ijms-24-03187-s001.zip › ijms-2141259-supplementary.pdf]

Supplementary Table S1. Comprehensive list of human psychiatric disorders and the number of publications using zebrafish as animal model.

Human psychiatric disorders (ICD-11 code)	Global prevalence	Zebrafish papers in PubMed
Neurodevelopmental disorders		**145**
6A02 autism spectrum disorder	1%	103 (autism spectrum disorder)
6A04 developmental motor coordination disorder	6%	3 (motor coordination disorder)
6A05 attention deficit hyperactivity disorder (ADHD)	7%	34 (ADHD)
Schizophrenia or other primary psychotic disorders		**32**
6A20 schizophrenia (schizoaffective disorder)	0.3%	31 (schizophrenia)
6A23 psychotic disorder	0.8%	1 (psychosis)
Mood disorders		**188**
6A60, 6A61 bipolar or related disorders	1%	21 (bipolar disorder)
6A70 depressive disorders	4%	167 (depression)
Anxiety or fear-related disorders		**675**
6B00 generalized anxiety disorder	7%	665 (anxiety)
6B01 panic disorder	13%	8 (panic)
6B04 social anxiety disorder	4%	2 (social anxiety)
6B20 obsessive-compulsive disorder (OCD)	1%	4 (OCD)
Disorders specifically associated with stress		**46**
6B40 post-traumatic stress disorder (PTSD)	4%	11 (post-traumatic stress)
QE84 acute stress reaction*	15%**	35 (acute stress)
Feeding or eating disorders		**5**
6B80 Anorexia nervosa	0.2%	5 (anorexia)
Disorders due to substance use or addictive behaviours		**243**
6C40 alcohol	18%	103 (alcohol-abuse/brain)
6C41 cannabis	4%	27 (cannabis)
6C42 synthetic cannabinoids	Unknown	2 (synthetic cannabinoids)
6C43 opioids	0.4%	10 (opioids addictive/abuse)
6C44 sedatives, hypnotics or anxiolytics	2%	3 (1 hypnotic, 2 anxiolytics)
6C45 cocaine	0.4%	37 (cocaine addiction or cocaine addictive)
6C46 stimulants	0.8%	1 (stimulants)
6C47 synthetic cathinone	1%	4 (synthetic cathinone)
6C48 caffeine	8%	19 (caffeine brain/abuse)
6C49 hallucinogens	4%	17 (hallucinogen),
6C4A nicotine	15%	5 (nicotine abuse/addiction),
6C4C MDMA	0.7%	8 (MDMA)
6C4D dissociative drugs	0.1%[##]	7 (ketamine)
6C7Z impulse control disorders	0.6 - 11%	**1** (impulse control disorders)
Disruptive behavior or dissocial disorders		**2**
6C90 oppositional defiant disorder	4% [#]	3 (oppositional defiant)
6C91 conduct-dissocial disorder	3% [#]	1 (conduct disorder)
Personality disorders and related traits		**1**
6D10 personality disorder	8%	1 (personality disorder)
Sleep-wake disorders		**157**
7A0Z insomnia disorders	10-30%	13 (insomnia)
7A2Z hypersomnolence disorders	6%	0 (hypersomnolence)
7A41 obstructive sleep apnea	12 %	0 (apnea)
7A6Z circadian rhythm disorders	3% [##]	144 (circadian rhythm)

*Does not belongs to disorders specifically associated with stress group.

**Prevalence of acute stress disorder following trauma depending on the nature and severity of trauma.

[#]Data from the National Research Council and Institute of Medicine (US).

[##]Data from the US population.
